# Constrained Geometry Organotitanium Catalysts Supported on Nanosized Silica for Ethylene (co)Polymerization

**DOI:** 10.3390/molecules22050751

**Published:** 2017-05-05

**Authors:** Kuo-Tseng Li, Ling-Huey Wu

**Affiliations:** Department of Chemical Engineering, Tunghai University, Taichung 407, Taiwan; vickyannyhot@gmail.com

**Keywords:** constrained geometry catalysts, titanium complex, silicas, nanoparticles, supports, ethylene polymerization and copolymerization, high density polyethylene, ethylene-α-olefin copolymers

## Abstract

Supported olefin polymerization catalysts can prevent reactor-fouling problems and produce uniform polymer particles. Constrained geometry complexes (CGCs) have less sterically hindered active sites than bis-cyclopentadienyl metallocene catalysts. In the literature, micrometer-sized silica particles were used for supporting CGC catalysts, which might have strong mass transfer limitations. This study aims to improve the activity of supported CGC catalysts by using nanometer-sized silica. Ti[(C_5_Me_4_)SiMe_2_(N^t^Bu)]Cl_2_, a “constrained-geometry” titanium catalyst, was supported on MAO-treated silicas (nano-sized and micro-sized) by an impregnation method. Ethylene homo-polymerization and co-polymerization with 1-octene were carried out in a temperature range of 80–120 °C using toluene as the solvent. Catalysts prepared and polymers produced were characterized. For both catalysts and for both reactions, the maximum activities occurred at 100 °C, which is significantly higher than that (60 °C) reported before for supported bis-cyclopentadienyl metallocene catalysts containing zirconium, and is lower than that (≥140 °C) used for unsupported Ti[(C_5_Me_4_)SiMe_2_(N^t^Bu)]Me_2_ catalyst. Activities of nano-sized catalyst were 2.6 and 1.6 times those of micro-sized catalyst for homopolymerization and copolymerization, respectively. The former produced polymers with higher crystallinity and melting point than the latter. In addition, copolymer produced with nanosized catalyst contained more 1-octene than that produced with microsized catalyst.

## 1. Introduction

Ethylene homo-polymers and copolymers (abbreviated as PE) are the most common polymers, accounting for about 38% of all the global plastics made today [1]. Their applications include bags, films, housewares, bottles, containers, pipe, tubing, wire and cable insulation, conduits and coatings [2]. About three quarters of the PE is produced via reactions catalyzed by transition metal catalysts, including Ziegler-Natta catalysts, metallocene catalysts, constrained geometry catalysts and supported metal oxides (Philips process) [1,3]. Metallocene catalysts and constrained geometry catalysts are single site catalysts, which can produce many new PE forms with enhanced properties [4].

Constrained geometry complexes (CGCs) are transition metal complexes containing linked monocyclopentadienyl amido ligand [5,6]. Group IV CGCs have been used as catalyst precursors for catalyzing olefin polymerization [4,5,6]. These catalyst precursors are inactive for olefin polymerization and must be activated with methylalumoxane (MAO) or B(C_6_F_5_)_3_. Compared to bis-cyclopentadienyl metallocenes, CGCs have less sterically hindered active sites and therefore have better ability for incorporating α-olefins, higher stability toward MAO, and are stable up to 160 °C [5].

Immobilized organometallic complexes can be used in gas–phase and slurry polymerization processes to overcome reactor fouling problems. Silica is the most commonly used support to immobilize single site catalysts [7,8], because it leads to polymer particles with good morphological features. In the literature, the sizes of silica particles used to support CGC catalysts were usually in the range of micrometers or above. For example, Kim and Soars [9] studied ethylene/1-hexene copolymerization at 40 °C and 50 °C using a constrained geometry catalyst (from Dow Chemical) supported on MAO treated silica (Grace Davison′s Silica 952, which is a micro-sized silica with a specific surface area of 300 m^2^/g and a pore volume of 1.65 cc/g [10]). For micro-sized catalysts, most active sites for polymerization are located inside fine pores (the average pore diameter of Davison silica is around 20 nm), and strong internal diffusion resistance might occur inside the pores. 

Nano-sized particles have very large external specific surface areas which can significantly reduce internal mass transfer resistance. Recently, we found that nano-sized silica supported metallocene catalysts (Cp_2_ZrCl_2_, Me_2_Si(Ind)_2_ZrCl_2_, and (C_2_H_4_)(Ind)_2_ZrCl_2_) had better activity than micro-sized silica supported metallocene catalyst for ethylene homopolymerization, copolymerization and for propylene polymerization [11,12,13,14,15].

In this study, we used a MAO-modified nanosized silica particle to support a constrained-geometry titanium catalyst, Ti[(C_5_Me_4_)SiMe_2_(N^t^Bu)]Cl_2_. We found that the nanosized catalyst system exhibited significantly better ethylene polymerization and copolymerization activity than a microsized catalyst system under identical reaction conditions. The maximum activities occurred at 100 °C, which is a higher temperature than that (60 °C) reported before for supported bis-cyclopentadienyl metallocene catalysts, and is lower than that (≥140 °C) used for unsupported Ti[(C_5_Me_4_)SiMe_2_(N^t^Bu)]Me_2_ catalyst. In addition, a FTIR study indicated that the copolymer produced with the nanosized catalyst contained more 1-octene than that produced with the microsized catalyst.

## 2. Results

### 2.1. Catalyst Characterization

**Figure 1:**
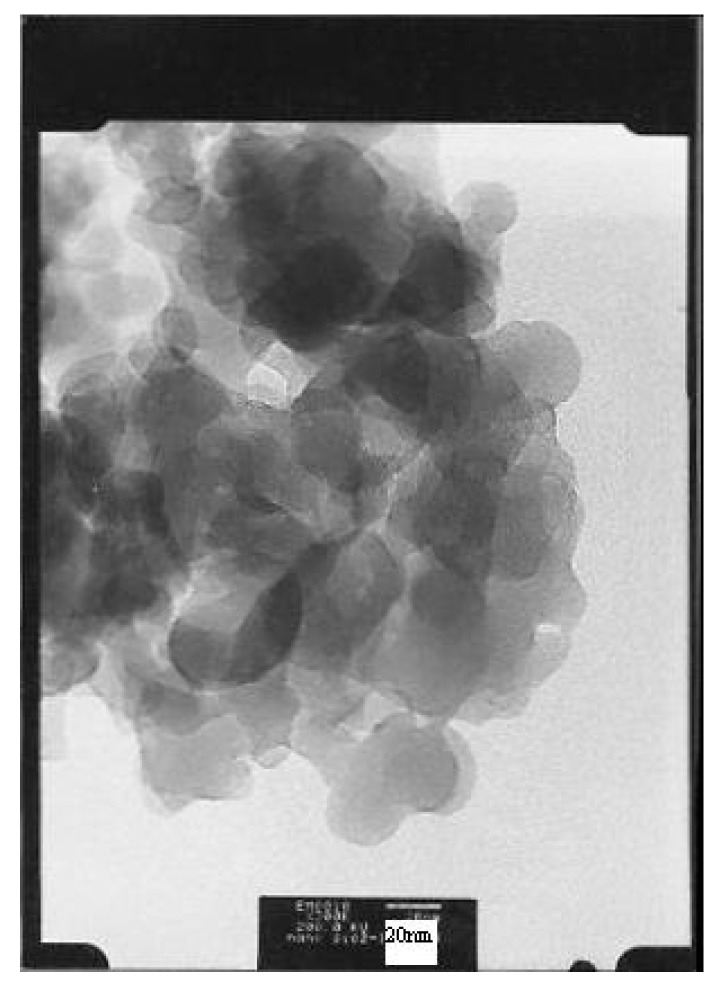
TEM picture of nanosized silica.

**Figure 2:**
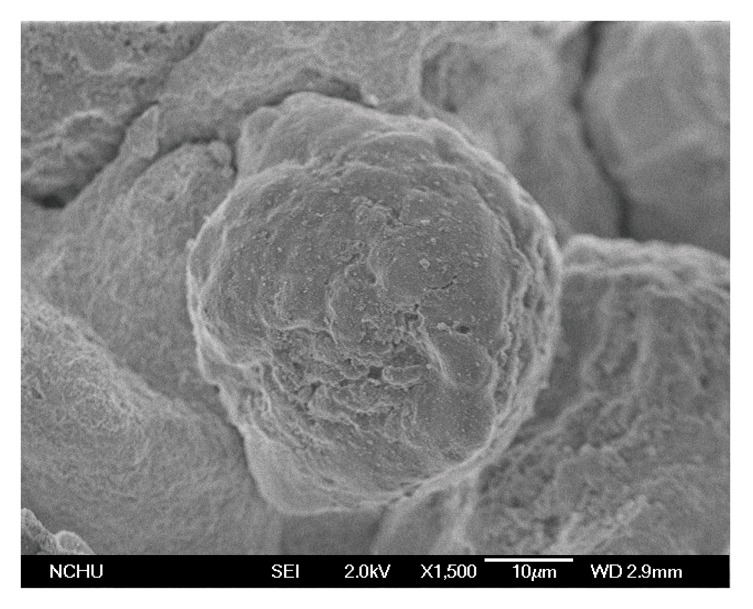
SEM photo of microsized silica.

MAO-treated nanosized and microsized silica were used to support Ti[(C_5_Me_4_)SiMe_2_(N^t^Bu)]Cl_2_. Nanosized silica particles have a size of 15–20 nm, as shown in the transmission electron micrograph (TEM) of Figure 1. Microsized silica particles have a diameter of greater than 40 μm, as shown in the scanning electron micrograph (SEM) of Figure 2.

Based on ICP-AES measurements for the supported catalysts, the Ti and Al contents of the nanosized catalyst were 0.121 and 10.5 wt % (i.e., [Al]/[Ti] ratio = 86.8), respectively, similar to those of the microsized catalyst (0.129 wt % Ti and 10.8 wt % Al (i.e., [Al]/[Ti] ratio = 83.7)). BET measurements indicated that surface area of nanosized slica was 640 m^2^/g, which was 2.1 times that of the microsized silica (surface area = 305 m^2^/g). The measurements of pore size distribution indicated that nanosized silica particles had very small pores (diameter ≤ 2.5 nm) and microsized silica particles had pores mostly in the 10–30 nm range (with an average pore diameter of 21 nm). MAO and Ti[(C_5_Me_4_)SiMe_2_(N^t^Bu)]Cl_2_ molecules can enter the large pores of microsized silica but cannot enter the tiny pores of nanosized slica, which resulted in the similar Ti and Al concentrations in these two catalysts, although the surface area of nanosized silica is about two times that of microsized silica.

### 2.2. Ethylene Homopolymerization and Copolymerization

**Figure 3:**
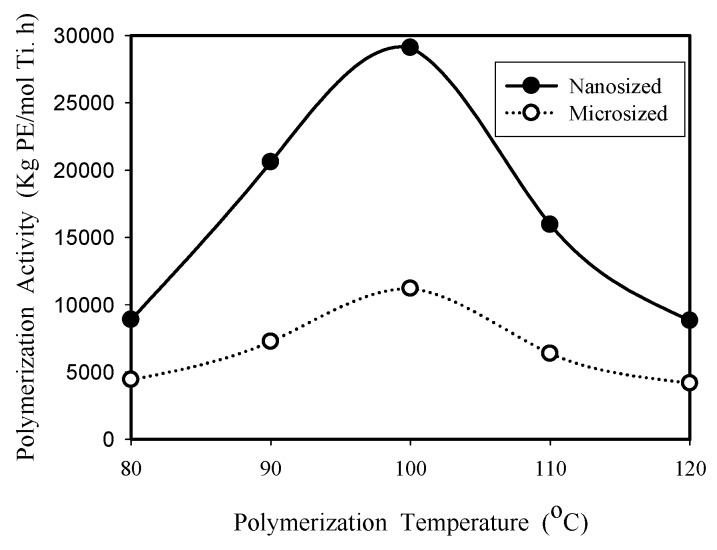
Influence of reaction temperature on homo- polymerization activity for nanosized and microsized catalysts.

**Figure 4:**
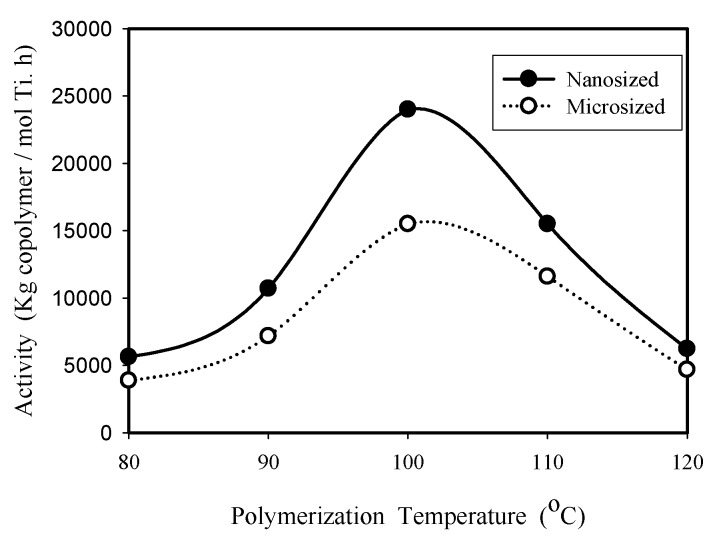
Copolymerization activity (ethylene with 1-octene) as a function of reaction temperature for nanosized and microsized catalysts.

The effect of polymerization temperature on the catalytic activity of nanosized and microsized catalysts was investigated in the temperature range of 80–120 °C with 1 h reaction time using 0.01 g catalyst in 100 mL toluene. Both ethylene homopolymerization and ethylene/1-octene copolymerization were studied, and the experimental results of polymerization activity as a function of reaction temperature are shown in Figure 3 and Figure 4, respectively. The optimum reaction temperature (100 °C) is higher than that (60 °C) reported before for supported bis-cyclopentadienyl metallocene catalysts containing zirconium atoms [12,13,15], which should mainly be due to the fact that titanium complexes are commonly less active than their zirconium analogues for the polymerization of ethylene alone or together with an α–olefin co-monomer [16]. In Figure 3, the maximum activities for the polymerization of ethylene alone are 29,100 kg PE/mol Ti.h and 11,200 kg PE/mol Ti.h for nanosized catalyst and microsized catalyst, respectively. In Figure 4, the maximum copolymerization activities obtained for nanosized catalyst and microsized catalyst are 24,400 kg copolymer/mol Ti.h and 15,500 kg copolymer/mol Ti.h, respectively. That is, the maximum activities of nanosized catalyst are 2.6 and 1.6 times those of micro-sized catalyst for homopolymerization and copolymerization, respectively.

It is interesting to note that nanosized catalyst had a higher homopolymerization activity than copolymerization activity (at 100 °C, the respective activities are 29,100 kg PE/mol Ti.h and 24,400 kg copolymer/mol Ti.h), while microsized catalyst had higher copolymerization activity than homopolymerization activity when T ≥ 100 °C (at 100 °C, the respective activities were 11,200 kg PE/mol Ti.h and 15,500 kg copolymer/mol Ti.h). This might be due to differences in active site location. Most active sites are located on the external surface of the nanosized catalyst, and most active sites are located inside the pores of the microsized catalyst. That is, the internal mass transfer resistance is more significant for the microsized catalyst, compared to that of the nanosized catalyst. Homopolymerization produces higher density polymers with higher crystallinity than copolymerization. Inside the pores of a microsized catalyst, it is easier for the reactants to diffuse through low density copolymer to reach the active sites than to diffuse through high density homopolymer, which results in the higher yield of copolymer than homopolymer for the microsized catalyst at the higher reaction temperature range. For nanosized catalyst, the diffusion effect is not so significant nor is the diffusion length so long because most active sites are located on the external surface, therefore, the amount of polymer produced is mainly determined by the intrinsic activity. Ethylene is more reactive than higher alkenes because the reported value of r_1_ = k_11_/k_12_ was 2.6 at 85 °C for Ti[(C_5_Me_4_)SiMe_2_(N^t^Bu)]Cl_2_/MAO catalyzed ethylene/olefin copolymerization [5].

Odian derived a rate expression for heterogeneous Ziegler-Natta polymerization using a Langmuir-Hinschelwood model [17], which can also be applied here. That is, the polymerization rate, R_p_, can be expressed as:

R_p_ = k_p_[C*] θ_M_ (1)

where k_p_ is propagation rate constant, [C*] is concentration of active species, and θ_M_ is the fraction of Ti surface covered by monomer, which is given by the following Langmuir isotherm:

θ_M_ = K_M_ [M]/(1 + K_M_ [M]) (2)

where [M] is the concentration of monomers in solution, and K_M_ is the equilibrium constant for monomer adsorption. Both K_M_ and θ_M_ decrease with the increase of reaction temperature because the enthalpy on the adsorption equilibria is exothermic. [C*] in Equation (1) is related to the polymerization time t [18]

[C*] = [C*] exp (−k_d_t) (3)

where [C*]_o_ is the initial concentration of catalytic active species, k_d_ is deactivation constant. It is also known that ethylene solubility in toluene ([M] in Equation (2)) decreases rapidly with increasing temperature [19]. Both the propagation rate in Equation (1) (k_p_ = k_p0_exp[−E_p_/RT]) and the catalyst deactivation rate in Equation (3) (k_d_ = k_d0_ exp[−E_d_/RT]) increase with the increase of reaction temperature. 

The volcano shape appearing in Figure 3 and Figure 4 can be explained in terms of the combined effects of reaction temperature on k_p_, [C*], and θ_M_ in Equation (1). In the temperature range of 80–100 °C, the increase of k_p_ with increasing temperature is greater than the decrease of [C*] and θ_M_, which results in an increase of polymerization activity R_p_. In the temperature range of 100–120 °C, the combined decrease of [C*] and θ_M_ with increasing temperature is greater than the increase of k_p_, which resulted in the decrease of polymerization activity, as shown in Figure 3 and Figure 4.

In Figure 3 and Figure 4, the polymerization activity of nanosized catalyst is more temperature sensitive than that of microsized catalyst, which also suggests that microsized catalyst has stronger diffusion resistance. It is known that true activation energy (close to nanosized catalyst) is equal to twice the apparent activation energy (close to microsized catalyst) when the internal diffusion resistance is strong [20].

The optimum reaction temperature (100 °C) observed for copolymerization in Figure 4 is different from those reported in the literature for unsupported CGC catalysts. The reaction temperature is usually run at temperature ≥ 140 °C for copolymerization of ethylene and 1-octene with unsupported Ti[(C_5_Me_4_)SiMe_2_(N^t^Bu)]Me_2_ catalyst (activated with B(C_6_F_5_)_3_) in solution process [1,21]. However, for the silica-supported Ti[(C_5_Me_4_)SiMe_2_(N^t^Bu)]Cl_2_ catalyst (activated with MAO) studied here, the catalyst activity decreased when reaction temperature was above 100 °C.

### 2.3. Polymer Characterization

**Figure 5:**
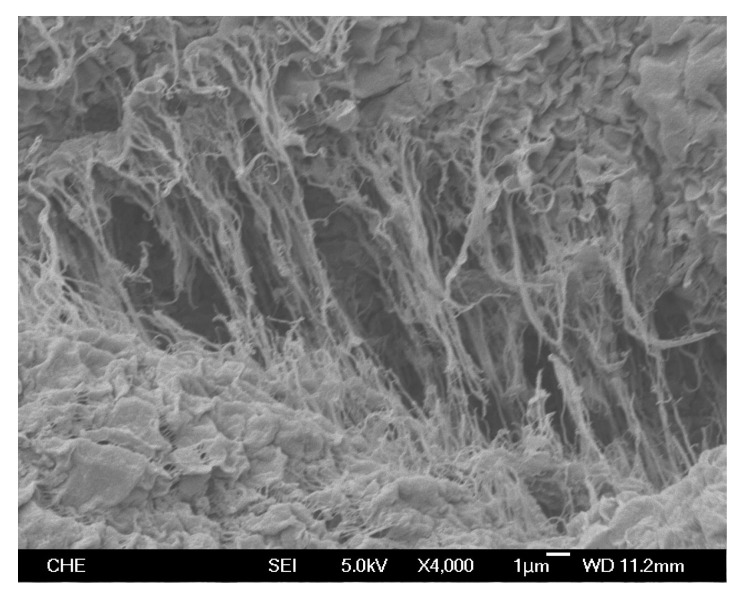
SEM photo of homopolymer produced with nanosized catalyst at 100 °C.

**Figure 6:**
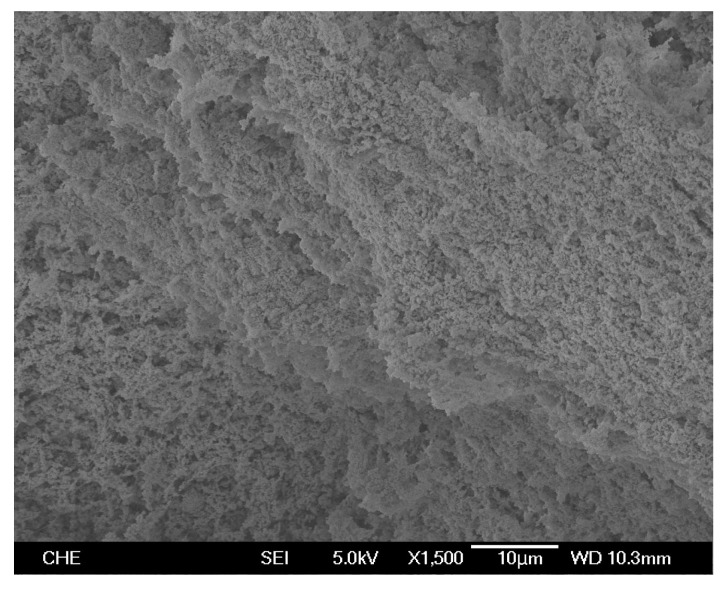
SEM photo of copolymer produced with nanosized catalyst at 100 °C.

SEM photos of the products from homopolymerization and copolymerization at 100 °C using nanosized catalyst are shown in Figure 5 and Figure 6, respectively. The morphology of homopolymer (shown in Figure 5) is very different from that of copolymer (shown in Figure 6). The phase structure of the homopolymer was mainly in two forms: discrete tiny flakes (~2.5 μm long, 1.5 μm wide) with long fibers (~15 μm long). In Figure 5, the fiber concentration is much higher and the fiber diameter is much larger than those observed before using a nanosized silica supported zirconocene catalyst (*rac*-dimethylsilbis(1-indenyl)zirconium dichloride) at 60 °C [15], which might be due to the difference of reaction temperature because only small amount of fiber was observed for homopolymer produced at 80 °C. The maximum amount of fiber occurred at 90–100 °C and then decreased with a further increase in reaction temperature. For HDPE produced with supported Ti[(C_5_Me_4_)SiMe_2_(N^t^Bu)]Cl_2_ catalyst, fiber and flakes are separated into distinct different regions, as shown in Figure 5. In previous zirconocene catalyzed polymerization, only a small amount of thin fibers dispersed in a predominantly particle-filled region was seen. The copolymer morphology (shown in Figure 6) is one of very uniform tiny particles with a size of less than 1 μm, which should be mainly the amorphous phase of copolymer. No fiber and flake structures appear in Figure 6, indicating that the fiber structure and flake structure are caused by crystallization of the polyethylene. 

Polyethylene is semi-crystalline and its density correlates well with crystallinity [2]. The measured density of homopolymer produced with nanosized catalyst at 100 °C is 0.964 g/cm^3^, which correspond to high density polyethylene (HDPE) because the historical definition of HDPE is the product of ethylene polymerization with density 0.94 g/cm^3^ and higher [2]. The polymer density produced with nanosized catalyst is higher than that (0.953 g/cm^3^) produced with microsized catalyst under the same reaction conditions. The measured density of copolymer produced with nanosized catalyst at 100 °C is 0.927 g/cm^3^, which corresponds to linear low density polyethylene (LLDPE) [2]. The copolymer density is also higher than that produced with microsized catalyst (0.914 g/cm^3^) under identical reaction conditions. The results indicate that the polymer chains produced with nanosized catalyst have more space to rearrange their molecules into more regular structures (i.e., higher density) than those produced inside the fine pores of a microsized catalyst.

**Figure 7:**
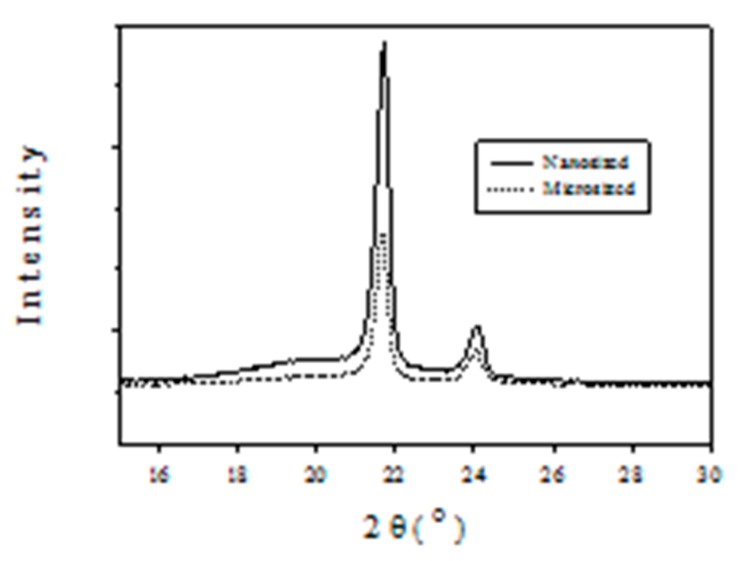
PE XRD patterns produced with nanosized and microsized catalysts at 100 °C.

**Figure 8:**
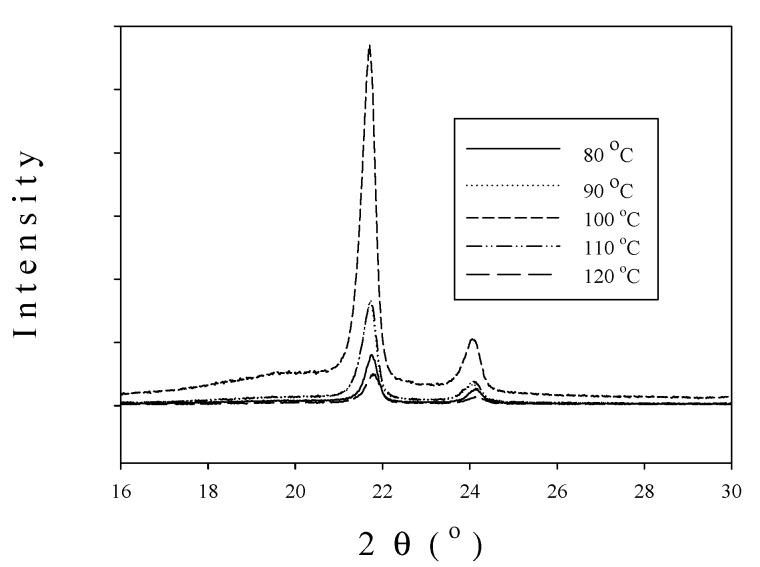
Influence of temperature on PE XRD patterns produced with nanosized catalysts.

Figure 7 compares the wide-angle X-ray diffraction (XRD) spectra of homopolymer obtained with nanosized and microsized catalysts at 100 °C, indicating that the former has much greater crystallinity than the latter because of the much higher intensity of the former. The effects of polymerization temperature on the XRD patterns of homopolymer produced with nanosized catalyst is shown in Figure 8, indicating that the peak intensity decreases in the following order: 100 °C >> 110 °C~90 °C >80 °C >120 °C, which is similar to the activity order reported in Figure 3.

The spectra in Figure 7 and Figure 8 exhibit the characteristic peaks of crystalline polyethylene at 2θ = 21.7° and 24.1°, which correspond to the reflection peaks of (110) and (200) planes, respectively [22]. The sharp peaks in Figure 7 and Figure 8 are due to the crystalline region scattering and the broad underlying ‘hump’ is due to non-crystalline region scattering [23]. Based on Figure 7, the crystallinity of polyethylene produced with nanosized catalyst is 66%, which is greater than that (53%) obtained with microsized catalyst.

**Figure 9:**
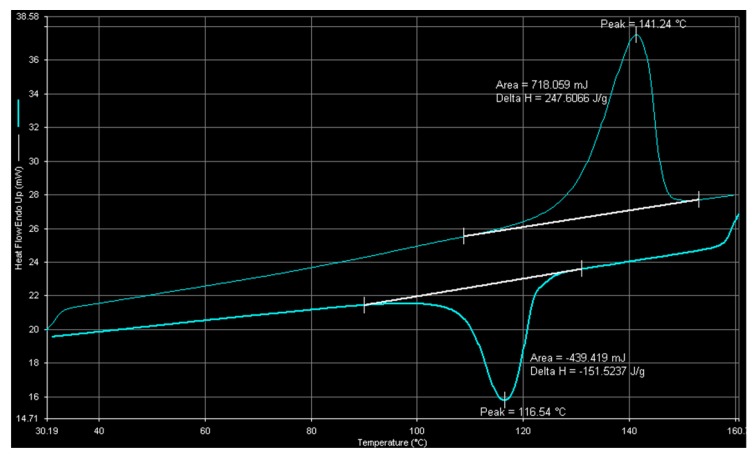
DSC spectrum of polyethylene obtained with nanosized catalyst at 100 °C.

**Figure 10:**
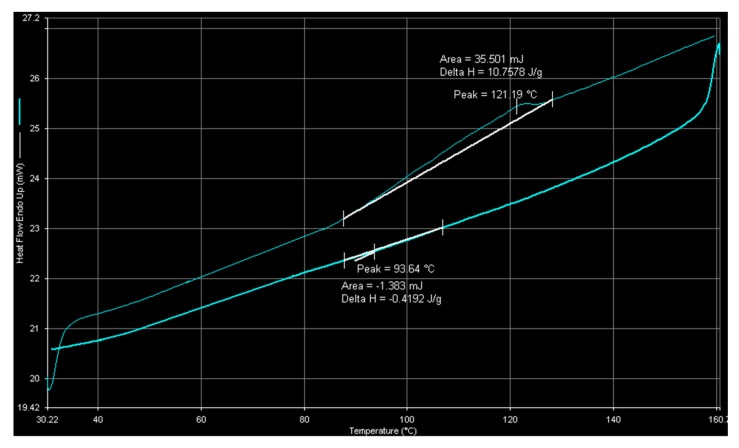
DSC spectrum of copolymer obtained with nanosized catalyst at 100 °C.

Differential scanning calorimetric (DSC) spectra of polyethylene and ethylene-1-octene copolymer obtained with nanosized catalyst are shown in Figure 9 and Figure 10, respectively. Figure 9 indicates that homopolymer produced with nanosized catalyst has a melting temperature and a crystallization temperature of 141.24 °C and 116.54 °C, respectively. The measured melting point is higher than that (140.08 °C) obtained with PE produced by microsized catalyst. Figure 10 indicates that ethylene-1-octene copolymer is almost completely amorphous (because of the small value of the heat of fusion) with a melting point of 121.2 °C. Based on the reported plot of melting point-density relationship for LLDPE [2], the sample of Figure 10 has a density of around 0.915 g/cm^3^ , which is lower than that (0.927 g/cm^3^) obtained by densimeter measurements (mentioned above). Number-average molecular weights of the samples in Figure 9 and Figure 10 were 6.0 × 10^4^ g/mol (with a PDI value of 2.0) and 5.3 × 10^4^ g/mol (with a PDI value of 2.2), respectively. The small PDI values indicate that the catalysts have uniform active sites.

**Figure 11:**
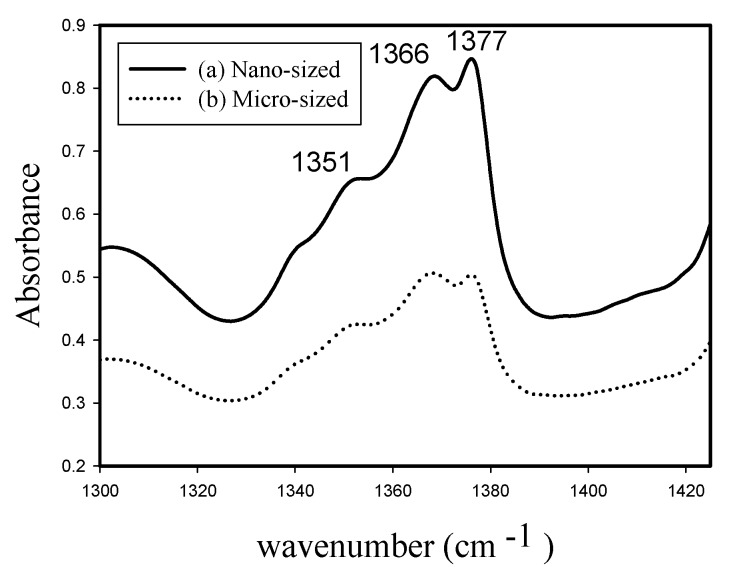
FTIR spectra (1300–1425 cm^−1^) of copolymers obtained with nano-sized and micro-sized catalysts at 100 °C.

Infrared spectroscopy is a powerful technique for studying the microstructure and determining the short-chain branch distribution of polyethylene [24]. Figure 11 compares Fourier-transform (FTIR) spectra (in the wave number range of 1300–1425 cm^−1^) of copolymers obtained at 100 °C for nanosized and microsized catalysts. Both spectra have three bands (at 1351, 1366, and 1377 cm^−1^). The bands at 1351 and 1366 cm^−1^ are assigned to methylene (CH_2_) wagging and the band at 1377 cm^−1^ is due to methyl (CH_3_) symmetric bending [25,26,27].

In Figure 11, the absorbance (y-axis) ratios of these three bands are different between spectra (a) and (b). For example, the absorbance ratios of the band at 1377 cm^−1^ to the band at 1366 cm^−1^ are 1.03 and 0.99 for spectra (a) and (b), respectively. Based on Beer’s law, absorbance is linearly proportional to concentration, therefore, Figure 11 indicates that copolymer produced with nanosized catalyst has more CH_3_ groups (i.e., more side-chain branches) than that produced with microsized catalyst. The difference of side chain group content should be due to the difference of reactant (ethylene and 1-octene) mass transfer resistance. 1-octene has lower diffusivity than ethylene, and is affected more significant by the stronger mass transfer resistance of microsized catalyst (compared to that of nanosized catalyst).

**Figure 12:**
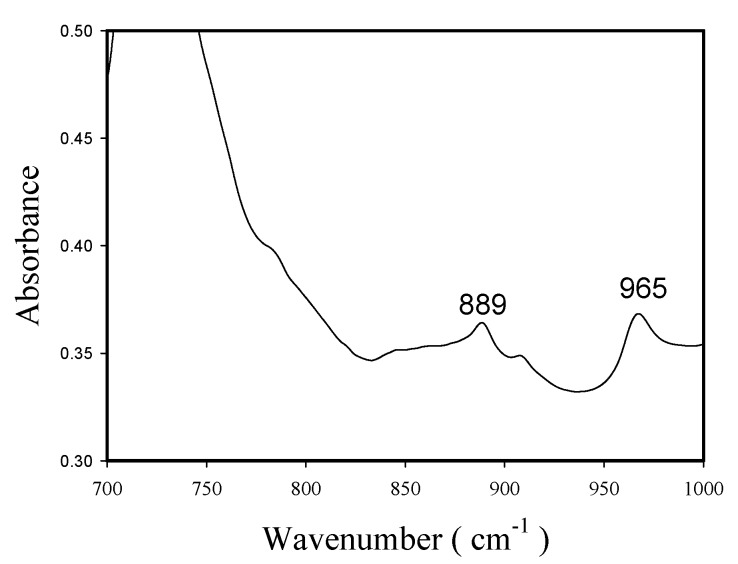
FTIR spectra (700–1000 cm^−1^) of copolymers obtained with nano-sized catalyst.

The sidechains of ethylene-1-octene copolymer are hexyl branches, which appear at the wave number of 888–889 cm^−1^ [28]. Figure 12 shows the FTIR spectra (in the wave number range of 700–1000 cm^−1^) of copolymer produced with nanosized catalyst. The peak at 889 cm^−1^ indicates that significant amounts of hexyl branching exist in the copolymer produced with nanosized catalyst. In Figure 12, the peak at 965 cm^−1^ might be due to *trans*-vinylene (-HC=CH-) groups [29].

## 3. Materials and Methods

Two silica sources were used to support CGC/methylaluminoxane (MAO) catalyst. One silica was nanosized, supplied by SeedChem (Melbourne, Australia); another was microsized, supplied by Strem Chemicals (Newburyport, MA, USA). The specifications of the microsized silica are almost identical to that of Grace Davison’s Silica 952, suggesting that they might be produced by the same manufacturing process. Ti[(C_5_Me_4_)SiMe_2_(N^t^Bu)]Cl_2_ and methylaluminoxane (MAO) (10 wt % solution in toluene) were supplied by Aldrich (St. Louis, MO, USA) and Albemarte (Baton Rouge, LA, USA), respectively.

Silica particles were calcined at 450 °C under a nitrogen flow (100 mL/min) for 4 h, then MAO was immobilized on the silica particles by heating 3 mL 10 wt % MAO solution (in toluene) with 0.5 g silica particle at 50 °C for 24 h, followed by washing with toluene three times to obtain MAO-treated silica. Ti[(C_5_Me_4_)SiMe_2_(N^t^Bu)]Cl_2_ (0.0115 g) was then reacted with the MAO-treated supports prepared above at 50 °C for 24 h under agitation, followed by washing with toluene three times, and drying. The above steps (except calcination) were carried out under a dry argon atmosphere by using glove-box techniques.

Catalytic polymerization of ethylene and copolymerization of ethylene with 1-octene were carried out in a 300-mL high-pressure autoclave reactor (supplied by Parr Instrument Co., Moline, IL, USA) equipped with an impeller and a temperature control unit. A thermo-regulated oven was used to heat the autoclave and a thermocouple was used to monitor the reaction temperature. In a typical experiment, 100 mL toluene, 5 mL MAO solution and 0.01 g supported CGC/MAO catalysts (prepared by impregnation method mentioned above) were charged to the reactor. The reactor was heated to the desired temperature (the temperature was set in the range of 80–120 °C). For copolymerization, 10 mL 1-octene was injected into the reactor. Ethylene at 200 psi was then introduced into the reactor to initiate the polymerization. Agitator speed was set at 400 rpm and the reaction time was 1 h. The polymerization was then terminated by adding acidic methanol and the polymer product was dried in a vacuum oven. The measured polymer weight was used for determining the polymerization activity according to the following equation: polymerization activity = (kilograms of homopolymer or copolymer)/(polymerization time × moles of Ti in the catalyst).

A scanning electron microscope (JSM-7000F, JEOL, Tokyo, Japan) was used to observe polymer particle morphology. An electronic densimeter (Mirage SD-120L, Alfa Mirage, Osaka, Japan) was used to measure polymer density. Polymer crystal structure was examined by X-ray diffraction (XRD) crystallography on an XRD-6000 diffractometer (Shimadzu, Kyoto, Japan) with Cu Kα radiation. The DSC measurements for the determination of the melting point, crystallization temperature, fusion and crystallization heats were carried out on a differential scanning calorimeter (Pyris-1, Perkin Elmer, Waltham, MA, USA). Each sample was melted at 160 °C and then cooled to room temperature to eliminate thermal history. Then, the melting endothermic curve was recorded with a heating rate of 10 °C/min, between 30 °C and 160 °C. A high-temperature gel permeation chromatography (Alliance GPCV-2000, Waters, Milford, MA, USA) with three columns (two Styragel HT6E and one Strragel HT2) at 135 °C were used to determine polymer molecular weight. A Shimadzu IR-Prestige 21 spectrometer was used for the acquisition of polymer film IR data.

## 4. Conclusions

The effects of silica particle size (nanosized and microsized) and polymerization temperature on a supported “constrained-geometry” titanium catalyst performances for the synthesis of high density polyethylene (HDPE; produced from ethylene homopolymerization) and linear low density polyethylene (LLDPE; produced from copolymerization of ethylene with 1-octene) have been presented. The optimum polymerization temperature for achieving the maximum activity occurred at 100 °C for both silica sizes and for both reactions, which was higher than that reported before for zirconcenes, but was lower than that (≥140 °C) used for unsupported Dow catalyst (Ti[(C_5_Me_4_)SiMe_2_(N^t^Bu)]Me_2_). Homopolymer morphology of the supported “constrained-geometry” titanium catalyst was different from that of zirconcenes, probably due to the difference of reaction temperature. At 100 °C, the nanosized catalyst had polymerization activities of 29,100 kg HDPE/mol Ti.h and 24,400 kg LLDPE/mol Ti.h, which were 2.6 and 1.6 times those obtained with microsized catalyst. XRD, DSC and density measurements indicated that the nanosized catalyst produced polymers with higher crystallinity, melting temperature and density than the microsized catalyst did, which might be due to differences in polymerization active site location (external for the former and internal for the latter). High temperature GPC measurements indicated that the nanosized catalyst had uniform active sites. FTIR studies indicated that the copolymer produced with nanosized catalyst contained more 1-octene than that produced with microsized catalyst.
